# Guidelines for a priori grouping of species in hierarchical community models

**DOI:** 10.1002/ece3.976

**Published:** 2014-02-22

**Authors:** Krishna Pacifici, Elise F Zipkin, Jaime A Collazo, Julissa I Irizarry, Amielle DeWan

**Affiliations:** 1 North Carolina Cooperative Fish and Wildlife Research Unit, Department of Applied Ecology, North Carolina State UniversityRaleigh, North Carolina, 27695; 2 Department of Zoology, Michigan State UniversityEast Lansing, Michigan, 48824; 3 U.S. Geological Survey, North Carolina Cooperative Fish and Wildlife Research Unit, Department of Applied Ecology, North Carolina State UniversityRaleigh, North Carolina, 27695; 4 RARE ConservationArlington, Virginia, 22201

**Keywords:** Biodiversity, community modeling, hierarchical modeling, imperfect detection, occurrence modeling, species richness

## Abstract

Recent methodological advances permit the estimation of species richness and occurrences for rare species by linking species-level occurrence models at the community level. The value of such methods is underscored by the ability to examine the influence of landscape heterogeneity on species assemblages at large spatial scales. A salient advantage of community-level approaches is that parameter estimates for data-poor species are more precise as the estimation process “borrows” from data-rich species. However, this analytical benefit raises a question about the degree to which inferences are dependent on the implicit assumption of relatedness among species. Here, we assess the sensitivity of community/group-level metrics, and individual-level species inferences given various classification schemes for grouping species assemblages using multispecies occurrence models. We explore the implications of these groupings on parameter estimates for avian communities in two ecosystems: tropical forests in Puerto Rico and temperate forests in northeastern United States. We report on the classification performance and extent of variability in occurrence probabilities and species richness estimates that can be observed depending on the classification scheme used. We found estimates of species richness to be most precise and to have the best predictive performance when all of the data were grouped at a single community level. Community/group-level parameters appear to be heavily influenced by the grouping criteria, but were not driven strictly by total number of detections for species. We found different grouping schemes can provide an opportunity to identify unique assemblage responses that would not have been found if all of the species were analyzed together. We suggest three guidelines: (1) classification schemes should be determined based on study objectives; (2) model selection should be used to quantitatively compare different classification approaches; and (3) sensitivity of results to different classification approaches should be assessed. These guidelines should help researchers apply hierarchical community models in the most effective manner.

## Introduction

Ecological communities are complex and many contain high levels of species diversity (Hubbell 2001), making it difficult to understand an individual species' role and/or contribution. Ecologists often classify communities to better understand the structure and function of individual species as well as the whole community. These classifications are determined by individual species' roles in a community, such as their functional similarity (ecosystem function, Picard et al. 2012), phylogenetic similarity (Ives and Helmus 2011), similarity in species' “ecological responses” to covariates (Ruiz-Gutiérrez et al. 2010) and environmental gradients (Dunstan et al. 2011, 2013; Ovaskainen and Soininen 2011; Jackson et al. 2012), or by similarities in specific species characteristics (e.g., sound intensity or sound pitch for aurally detected species; Alldredge et al. 2007).

Previous studies have relied on expert knowledge to classify species (Gitay et al. 1999), statistical clustering methods (Xu and Wunsch 2005), model-based clustering methods (Dunstan et al. 2011, 2013), and multilevel or hierarchical models to group species (Dorazio and Royle 2005; Ives and Helmus 2011; Ovaskainen and Soininen 2011; Jackson et al. 2012). A unique feature of hierarchical community models is the ability to incorporate sampling errors as well as explicitly account for individual species traits (Dorazio and Royle 2005; Dorazio et al. 2006). These approaches are developed using species occurrence models (MacKenzie et al. 2006), which are then linked together within a hierarchical framework (Royle and Dorazio 2008). Hierarchical community models are generally used to: (1) estimate species richness; (2) test hypotheses about community-level responses to perturbations (e.g., environmental covariates, or management/conservation actions); and (3) estimate habitat or covariate parameters for individual species.

Hierarchical multispecies models “borrow” information across all species in a community, which leads to more precise species-level inferences (Dorazio and Royle 2005). In such models, each species influences the parameter estimates of all other species in the community (or group within the community, depending on the exact hierarchical structure of a model). As a result, individual species-level estimates are a combination of the single species and the average estimate of those parameters for the entire community (or group of species). The degree to which estimates are pooled together rather than estimated separately (i.e., pooling or “shrinkage”) is dependent upon the quality and quantity of available data (e.g., number and locations of species detections; Gelman and Hill 2007). A major benefit of shrinkage is the ability to estimate parameters for species that are rarely detected and would otherwise not be estimable or would be too imprecise for meaningful inference (Kéry and Royle 2008). Multispecies hierarchical models have subsequently been used to address community-level responses to environmental factors (Zipkin et al. 2009; Burton et al. 2012; Jones et al. 2012) and management activities (Russell et al. 2009; Zipkin et al. 2010; Giovanini et al. 2013; Hunt et al. 2013) as well as understanding individual species-level responses to landscape/habitat features (Tingley and Beissinger 2013) and management or conservation actions (Grant et al. 2013; Sauer et al. 2013). The approach is also particularly useful for rare or infrequently detected species (White et al. 2013).

The question of how to best group species and the implications of such groupings has not been thoroughly explored and represents an important step in ensuring the most appropriate use of hierarchical multispecies models. Our objectives were to evaluate the influence of different a priori classification approaches on two data sets. Specifically, we are interested in assessing the sensitivity of community- and species-level inferences to various classification schemes for grouping species assemblages. We explore the implications of these groupings on parameter estimates for avian communities in two different ecosystems: (1) urbanized, agricultural, and forested landscapes within two forest reserves of southwestern Puerto Rico and (2) temperate forests within the northeastern United States. We report on classification performance and the variability in species- and community-level inferences that is observed depending on the classification scheme. We also provide guidelines about how to carefully apply these models and the potential trade-offs of different grouping schemes.

## Materials and Methods

### Study Area – Puerto Rico

The Puerto Rico study area is in southwestern Puerto Rico and consists of a matrix of habitat located between the Guánica and Susuá Forest Reserves. The study area was divided into three identifiable and dominant (at least 70% coverage) habitat types: forest (5536 ha), urban (6116 ha), and agriculture (7066 ha). A total of 128 point count sites were randomly established across the three habitat types and in the Guánica and Susuá Forest Reserves. Thirty sites were in each of the forest, urban, and agriculture habitat types for a total of 90 sites. An additional 38 sites were established between 300 and 1500 m within the Guánica (18 sites) and Susuá (20 sites) Forest Reserves and these are referred to as edge habitat. Survey sites within agriculture and urban habitat types were at least 500 m apart and those in the forest habitat type and “edge” habitat were >1 km apart to minimize correlation among sites. Survey sites between different habitat types were at least 1 km apart. Avian surveys were conducted during the breeding season (March–June) of 2010 and 2011 by trained observers and consisted of three repeat visits to each site in each year. All resident avian species detected by sight or aurally during a 10-min period were recorded. For more detailed information about the study area and sampling design refer to Irizarry (2012).

### Study Area – Hudson River Valley

The temperate forest study area is located in the Hudson River Valley (HRV), New York, which is a 954,600 ha region, north of New York City. A total of 72 point count sites were randomly selected in deciduous and mixed-deciduous forest fragments across the HRV, New York. Avian surveys were conducted from 15 May to 1 July of 2006 and 2007. Two trained observers recorded all species seen or heard during the 10-min, 250-m fixed-radius point counts at each sampling site. Sites were visited on three separate occasions during the breeding season (once each per 2-week period), although not all sites were surveyed both years. Three covariates thought to influence the breeding success of birds were also recorded at each location: the forest fragment area in which the site occurred, the perimeter of the fragment, and perimeter/area ratio (P/A). For more details about the sampling design and study area refer to DeWan et al. (2009).

### Modeling framework

We modeled both communities using the approach of Dorazio and Royle (2005) and Dorazio et al. (2006). The same general model was fit to both data sets (but covariates differed between data sets, see below) and for the different groups within each classification scheme (Table 1). Let *N* denote the unknown number of unique species that occur within the region of interest (here *N* can represent the total community of species or the number of species within a group). Surveys are conducted wherein each of the *j = *1,2*,…,J* sites is visited *k = *1,2*,…,K* times, and the identities of all species *i *=* *1,2,*…,n* are recorded as they are detected during the sampling event. We assume that the total number of surveys *K* are conducted within a sufficiently short period of time such that *N* remains constant (i.e., community closure).

**Table 1:** The different classification approaches and the associated groups for the Puerto Rico and Hudson River Valley data sets.

Puerto Rico		
Habitat	Microhabitat	Diet
H1: Wet, upper forests	M1: Dense forest	D1: Carnivore
H2: Open/anthropogenic	M2: Grassland	D2: Frugivore
H3: Coastal forest	M3: Interspersed forest	D3: Granivore
	M4: Open	D4: Insectivore
		D5: Nectarivore
		D6: Omnivore
Hudson River Valley		
Nest location	Habitat	Diet
N1: Bush nest	H1: Arboreal	D1: Frugivore
N2: Cavity	H2: Bush	D2: Granivore
N3: Ground nest	H3: Ground/shrub	D3: Insectivore
N4: Ledge	H4: Open air	D4: Omnivore
N5: Tree nest	H5: Terrestrial	
	H6: Tree trunk	
	H7: Vegetation	

Site-specific occurrence for species *i *=* *1,2,…*n*,…,*N* at site *j*, denoted *z*_*ij*_, is a latent random variable where *z*_*ij*_ = 1 if species *i* occurs in site *j* and is zero otherwise. We specify the occurrence model as *z*_*ij*_ ∼ Bern (ψ_*ij*_) where ψ_*ij*_ is the probability that species *i* is present at *j*. True occurrence is only partially observed through the detection/nondetection data where *x*_*ijk*_ (recorded as a 1 if a species is observed and zero otherwise) for species *i* at site *j* during sampling period *k* is *x*_*ijk*_ ∼ Bern (*p*_*ijk* *_ z_*ij*_). The parameter *p*_*ijk*_ is the detection probability of species *i* at site *j* for the *k*th sampling period. If species *i* is present (*z*_*ij*_ = 1) at site *j,* then the probability of detecting that species is *p*_*ijk*_ otherwise if *z*_*ij*_ = 0, then *x*_*ijk*_ = 0, and we ensure that detection is a fixed zero when a species is not present.

We incorporated covariate effects into the occurrence and detection models linearly on the logit-probability scale. For the PR data set, we modeled occurrence as a function of the site-level habitat type (agriculture, forest, edge, and urban) on the logit scale as follows: logit (ψ_*ij*_) = *μ*_*i*__,habitat(*j*)_, where *μ*_*i*__,habitat(*j*)_ is the occurrence probability (on the logit scale) for species *i* in the habitat type of location *j* (i.e., forest, urban, agriculture, or edge). The detection probability of species *i* in PR was assumed to vary based on the year of the survey. We assumed that the community was closed (i.e., the species pool remained constant) over the 2 years during which the survey was conducted, but added a year effect (constant across species) to account for changes in detection between the 2 years as a result of annual fluctuations in seasonality: logit (*p*_*i*_) = *υ*_*i*_* + β*_*year*_.

We followed the model described in Zipkin et al. (2009) for the HRV data set where habitat covariates (fragment area, perimeter, and perimeter/area ratio [P/A]) were assumed to influence species occurrences as follows: logit (ψ_*ij*_) = *μ*_*i*_* *+ *α*1_*i*_ perimeter_*j*_* +* *α*2_*i*_ area_*j*_ + *α*3_*i*_ *P/A*_*j*_. We similarly modeled the detection probability for species *i* as a function of survey date (linear and quadratic effects) and the year of the survey: 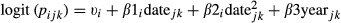. All covariates were centered and normalized (mean = 0, variance = 1) such that the inverse logit of *μ*_*i*_, for example, is the occurrence probability for species *i* in sites with “average” habitat conditions. Again we assumed that the community was closed (i.e., the species pool remained constant) over the 2 years during which the survey was conducted and used the year effect (constant across species) to account for changes in detection between the years.

### Classification and grouping of species

Our interest lies in comparing different classification approaches that could be used to group species and the associated community- and species-level inferences for each approach. To this end, we made use of several common approaches to classify species for both the PR and HRV data sets. We focused on traits that would influence occurrence probability or species richness and did not explore how choice of grouping affected inferences on detection but analogous approaches could be used to do so.

For both data sets, we identified grouping/classification approaches that correspond to the three general uses of hierarchical models (i.e., species richness, community-level effects, and species-level effects) and potentially resulted in different responses with respect to the covariates of interest. First, we wanted to explore groupings based on large or coarse scale habitat requirements. For the PR data set, we grouped species according to their dominant land-cover associations, and for the HRV data set, we grouped species according to their foraging habitat associations (Hamel 1992; Poole 2005). Large-scale habitat features are responsible for identifying community-level responses to environmental stressors (e.g., fragmentation, urbanization) as well as understanding attributes of biological integrity that would be manifested in species richness (Caro 2010). For example, in Puerto Rico, we hypothesized that the forest matrix of habitat would support greater species richness and occurrence of native species than the urban and agricultural landscapes because resident avifauna evolved in forested landscapes (Lugo et al. 2012). Second, we used a smaller scale of habitat preference, one that pertains to the use of various habitat components within large-scale habitat classes (e.g., feeding, cover, nesting substrates). This grouping reflects the purported hierarchical nature of habitat selection wherein local-scale habitat features drive species occurrence and composition (Johnson 1980). For the PR data set, we used categories that reflected the availability of habitat for feeding and cover (microhabitats). For the HRV data set, we used the dominant forest type for the preferred nest location (nest location; Hamel 1992; Poole 2005). Finally, we used the predominant diet of individual species to classify species in both the PR and HRV data sets. Diet is expected to influence the spatial distribution of species within habitats because individuals will be more selective at a finer scale driven by habitat structure and composition, dietary needs and prey availability (Robinson and Holmes 1982; Wood et al. 2012). For example, in PR, we expect that frugivore occurrence and richness would be particularly sensitive to urbanized and agricultural habitat patches because the distribution and quantity of fruiting resources is vastly different than that of forested environments (Irizarry 2012). In the HRV data set, most birds eat insects during the breeding season, but it is known that their diet is broader and we categorized species accordingly (Table S1). For example, in HRV, we expect that patch area and perimeter size would influence the occurrence of insectivores and omnivores because their foraging ability depends heavily on the availability of prey (Robinson and Holmes 1982; Wood et al. 2012).

An additional hierarchical component was added to the model wherein we assumed that the species-level parameters were random effects governed by community- or group-level hyperparameters. All coefficients from the models in both data sets (PR and HRV) were assumed to come from a normal distribution (e.g., *α*1_*i*_* *∼* N* (*μ*_*α*1_, *σ*_*α*1_)) where the mean of the distribution represents the community or group response to that particular covariate and the standard deviation is the variation among species within the particular group. We follow Dorazio et al. (2006) and use a parameterization of the unconditional likelihood and data augmentation to estimate species richness *N* for all groups within each of the different classification schemes.

We fit each of the models with the different classification schemes separately using a Bayesian approach in WinBUGS (Spiegelhalter et al. 2003) through R (R2WinBUGS; Sturtz et al. 2005) by running three parallel chains each of length >77,000 with burn-in of at least 25,000 and thinning by 25 (code available in Data S1). We used vague priors (e.g., uniform distribution from 0 to 1 for community- or group-level occurrence and detection covariates; normal distributions with mean zero and variance 1000 for community- or group-level habitat and sampling covariates) for all hyperparameters in all models. Convergence was confirmed through trace plots, correlation, and the Gelman–Rubin statistic (Gelman and Rubin 1992).

### Model evaluation

We used area under the receiver operating characteristic (AUC) curve to evaluate the model fit of each classification scheme (Sing et al. 2005; Fawcett 2006). In the context of occurrence models, AUC measures a model's goodness of fit by estimating the probability that a randomly chosen occupied sampling point (*z*_*ij*_ = 1) has a higher occurrence probability than a randomly chosen unoccupied sampling point (*z*_*ij*_ = 0). If a model fits well, then it consistently predicts a higher occurrence probability for occupied sites yielding an AUC closer to 1.0. If a model performs poorly, it will perform the same as chance yielding an AUC closer to 0.5 (Fawcett 2006).

W calculated the mean and 95% Bayesian credible intervals (BCI) AUC values reflecting goodness of fit for each of the classification approaches for both data sets using an approach that accounts for the fact that *z*_*ij*_ is a latent variable and thus can only be imperfectly observed (Zipkin et al. 2012; Mattsson et al. 2013). This provided an overall AUC value for each model classification scheme that allowed us to evaluate relative model fit for each classification approach in both data sets.

## Results

**Figure 1:**
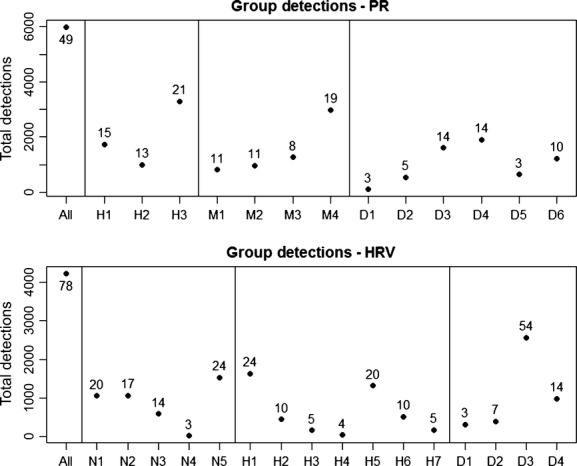
Group summaries (total number of species and detections for each group) for the different classification schemes associated with the Puerto Rico and Hudson River Valley data sets. We used three different classification approaches for each data set; PR: H – Habitat, M – Microhabitat; D – Diet; Hudson River Valley: Nest location – N, Habitat – H, D – Diet, see Table 1.

Forty-nine species were detected during the PR study with a total of 5998 detections. Group size ranged from three species with only 98 total detections (Diet group 1; carnivores) to 23 species with 3287 detections (Habitat 3; coastal forest; Fig. 1). Individual species detections ranged from 2 detections (cave swallow; *Petrochelidon fulva*) to 556 detections (bananaquit; *Coereba flaveola*; Fig. S1). The model that grouped all species together had the highest mean AUC value (mean: 0.80; BCI: 0.76–0.85) followed by the Habitat (mean: 0.75; BCI: 0.68–0.82), Microhabitat (mean: 0.74; BCI: 0.65–0.82), and Diet (mean: 0.71; BCI: 0.54–0.82) models. However, BCIs were overlapping indicating uncertainty about which classification scheme fit the PR data best.

Seventy-eight species were detected during the HRV study with a total of 4200 detections. Group size ranged from three species with only 14 total detections (Nest 4; ledge group) to 54 species with 2570 detections (Diet 3; insectivore; Fig. 1). Individual species detections ranged from 1 detection (Eastern bluebird, *Sialia sialis*; magnolia warbler, *Dendroica magnolia*; Nashville warbler, *Vermivora ruficapilla*; pine siskin, *Carduelis pinus*) to 239 detections (black-capped chickadee; *Poecile atricapillus*; Fig. S1). The all species grouped together model (mean: 0.84; BCI: 0.77–0.9) also had the highest mean AUC for the HRV data set, followed by the Diet (mean: 0.79; BCI: 0.57–0.93), Habitat (mean: 0.77; BCI: 0.54–0.96), and Nest location (mean: 0.75; BCI: 0.52–0.91) models. Again, BCIs for all schemes where overlapping.

### Species richness

**Figure 2:**
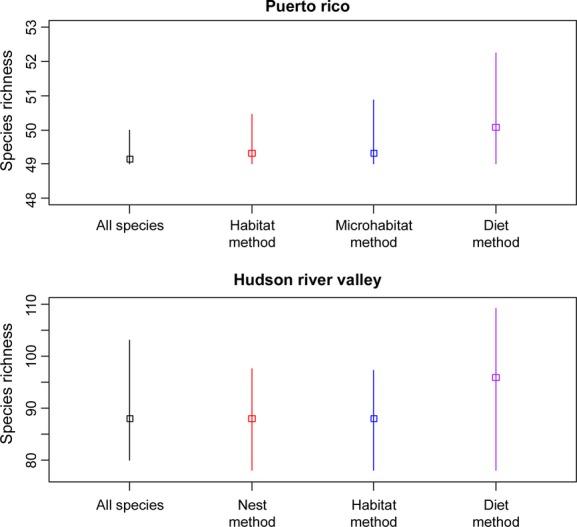
Posterior mean estimates of species richness with 95% posterior credible intervals for the full community model and each of the three different classification approaches for the Puerto Rico (top) and Hudson River Valley (bottom) data sets.

Estimates of species richness for the PR data set were very similar across all classification schemes and ranged from 49.15 to 50.08 (posterior means; Fig. 2). The BCI on species richness was largest for the diet classification scheme, but was still relatively narrow (49–52; Fig. 2). Estimates of species richness by site were consistent among all classification schemes with the diet groupings showing the largest amount of uncertainty (Fig. S2).

Estimates of species richness for the HRV data set ranged from 87.91 (posterior means: all species) to 95.89 (Diet), and all three classification schemes produced estimates that were higher than estimates from the full community model (i.e., all species combined; Fig. 2). The BCI was largest for the Diet scheme and relatively similar for the other approaches. Estimates of site-level richness were higher for all of the classification schemes compared with using all species together (Fig. S2). Similar to the PR results, the Diet scheme had the most variability around each of the site-level estimates of species richness (Fig. S2).

### Community-level inference

**Figure 3:**
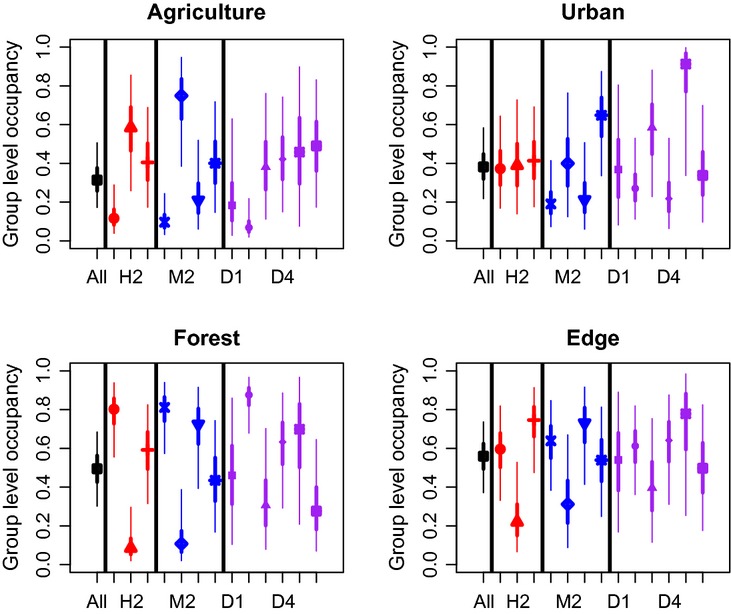
Community- and group-level mean occurrence probabilities (posterior means with 95% and 50% Bayesian credible intervals) in the four different habitat types (agriculture, forest, urban, and edge) in the southwestern Puerto Rico study area. We used three different classification approaches to group species: Habitat – red, Microhabitat – blue, and Diet – purple, along with using all of the species – black, see Table 1 for more details.

Community or group-level occurrence estimates were consistent among classification schemes in response to the urban and edge habitats for the PR data set (Fig. 3). There was much larger variability in how groups responded to both agriculture and forest habitat (Fig. 3). For example, the open/anthropogenic group in the habitat classification, the grassland group in the microhabitat classification, and the frugivore group in the diet classification tended to show unique responses that significantly differed from other groups including when all species were combined. There was no consistent pattern in the relationship between total number of detections or number of species within a group to performance (in terms of precision of parameter estimates). In some instances, groups with small sample sizes and/or number of total species had very different responses to habitat and high levels of within-group uncertainty, but this was not always the case. For example, the diet approach had three groups with <5 species and the lowest number of total detections, but in some instances, these groups showed the lowest within-group variability (Diet 2; frugivores) in response to agriculture and forest habitats, leading to more precision in species-level estimates.

**Figure 4:**
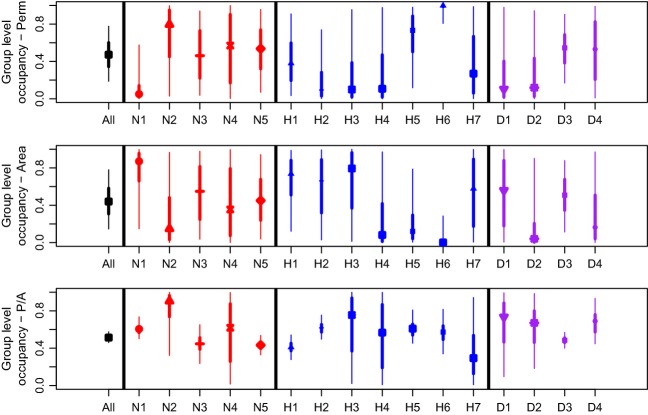
Community-level effects on the occurrence probability (posterior means with 95% and 50% Bayesian credible intervals) to three different covariates, perimeter (Perm), area (Area), and perimeter/area ratio (P/A) for the Hudson River Valley data set. We used three different classification schemes to group species: Nest – red, Habitat – blue, and Diet – purple, along with using all of the species – black, see Table 1 for more details.

Community or group-level parameters for the HRV data set tended to have larger variances compared with the PR data (Fig. 4). Results were most consistent for group-level responses to P/A where the all species approach had the lowest variability (Fig. 4). The response to perimeter and area was not as clear as there was evidence of substantial variation among almost all of the group responses (Fig. 4). For example, the bush nesters (group 1) had a negative response to perimeter, while the tree trunks habitat group (6) showed a positive response to perimeter compared with the all species combined group, which showed a slightly negative response (Fig. 4). Such patterns were found for other covariates as well, suggesting that groups of species respond very differently to habitat covariates. Not surprisingly, these differences tend to be “averaged” out when using all of the data in one community-level grouping.

Group-level responses also show a large amount of variation for the HRV data set (Fig. 4). Several groups have wide credible intervals, (e.g., ground/shrub, open, tree trunk groups – 3, 4, and 6, respectively, and frugivores and granivores – Diet groups 1 and 2), suggesting that there is a lot of within-group variability not strictly due to low sample sizes (sample sizes range from 31 for the open habitat group to 508 for the tree trunk habitat group). Similar to the PR data set, there was no strict relationship or pattern between sample size and group performance. In most instances, groups with a higher sample size had narrower BCIs, but there were many exceptions (e.g., bush group, H2, in response to P/A).

### Species-level inference

**Figure 5:**
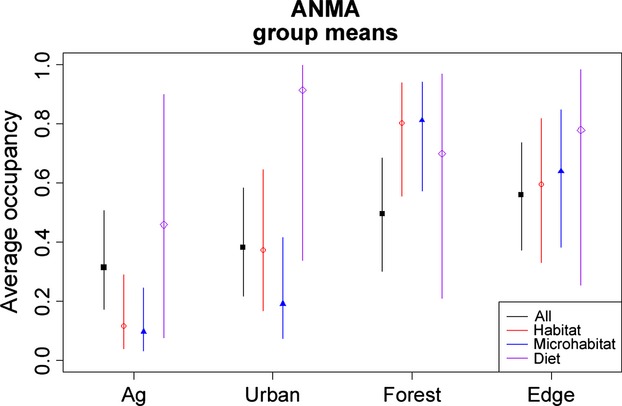
Community-level mean occurrence probabilities in the four different habitat types (agriculture, forest, urban, and edge) for the groups that the Antillean mango (ANMA, *Anthracothorax dominicus*) was classified in including the wet, moist upper forests (habitat classification), dense forests (microhabitat classification), and frugivores (diet classification) groups.

For the PR data set, species-level responses to habitat were consistent among the different classification approaches with overlapping BCIs (e.g., Fig. S3). Figure 5 shows the group response for each of the groups that Antillean mango (ANMA; *Anthracothorax dominicus*) belongs to in relation to habitat. Species with fewer detections generally had more variability in their individual estimates regardless of the group they were in, with few exceptions.

**Figure 6:**
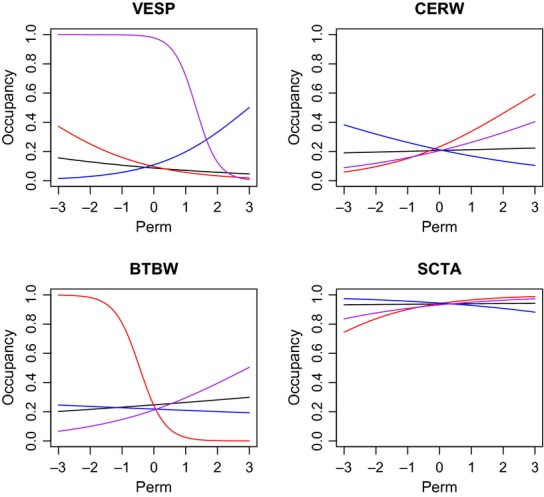
Mean marginal occurrence probabilities for four priority conservation species (Vesper sparrow, VESP: two detections at two sites; cerulean warbler, CERW: seven detections at six sites; black-throated blue warbler, BTBW: 20 detections at nine sites; and Scarlet tanager, SCTA: 141 detections at 56 sites) in relation to forest fragment perimeter in the Hudson River Valley. We explored three different classification schemes for grouping species: Nest location – red lines, Habitat – blue lines, and Diet – purple lines; black lines represent using all of the species. Covariates have been standardized to have a mean of zero and variance of one. Although uncertainty was high for all models, using all species had the lowest uncertainty (narrowest credible intervals) followed by nest location, habitat, and diet models.

Species-level occurrence estimates for the HRV data set also had BCIs that overlapped among the different classification schemes in all instances. However, in many cases, individual species had occurrence estimates who's BCIs ranged from 0 to 1 (Fig. S5). Marginal mean response to covariates often varied significantly leading to very different predicted responses for certain species based on classification. This result was closely tied to sample size and the specific covariate of interest (Fig. 6 and Fig. S4). Species inferences can be extremely variable depending on the classification approach when the number of detections is low (e.g., Vesper sparrow, *Pooecetes gramineus*), but this was not always the case (e.g., cerulean warbler, *Setophaga cerulean*; Fig. 6). Species with low sample sizes generally had wider BCIs and more discrepancy in individual estimates among groups, suggesting that species-level inferences for rare species should be approached with caution. However, these problems diminished as the number of detections increased.

## Discussion

We showed that different classification schemes have the potential to affect inference at both the community- and species-level, while species richness appeared to be the metric that was most robust to differences in group classifications. In both data sets, using all species in one group (i.e., the full community model) resulted in the most precise estimates (smaller variability in species richness BCI) and arguably, the best model fit as measured by AUC although many of the AUC BCIs overlapped. However, we also found that there were often unique group-level responses to habitat covariates that were missed when species were all grouped together because these distinct responses were “averaged out” (Sauer and Link 2002). The sensitivity of inference to grouping at both community and species levels is a clear indication that the specific motivations for using multispecies models should be articulated and that this should precede any data collection and/or analysis.

In both data sets, using all species in one group (i.e., the full community model) resulted in the most precise estimates, while the Diet group appeared to have the greatest uncertainty (largest credible intervals) around estimates of species richness. This uncertainty was even more dramatic in the HRV data set where the posterior mean was significantly higher for the Diet group compared with any of the other schemes including all of the species analyzed together. This may be due to a smaller total sample size for the HRV data set compared with the PR data set. A second hypothesis is that the Puerto Rican species are less sensitive to grouping because they are much more adept at utilizing different habitats. Strictly defining species groups may not be particularly meaningful in many instances because island species are capable of enduring landscape changes and disturbances (e.g., hurricanes), and exploiting novel resources (Lugo et al. 2012).

We expect similar complications will arise in many studies when it is difficult to clearly define species' groups or there are many factors influencing species habitat preferences. Often the underlying ecological mechanisms that regulate species' responses are not understood (usually this is the motivation for research), yet this information is critical in classifying groups of species. It is also possible that species are responding to different covariates based on more than one classification scheme. For example, shrub cover could be important to ground nesters, but mature trees could be important to granivores and one species may belong to both the ground nesters and granivores. Rare species present unique challenges because inferences are further compounded by scarce data. They typically require extra sampling effort just to collect a sufficient amount of data for analysis (Stockwell and Peterson 2002; Thompson 2004; Noon et al. 2012). We found that the estimated response of data-poor species can be highly variable depending on the grouping approach and associated group members (e.g., Fig. 5). This suggests that caution should be taken when interpreting model results for species with few detections and ideally studies should be explicitly designed to obtain sufficient data for rare species if this is the main objective (Thompson 2004; Pacifici et al. 2012).

We suggest the following guidelines for the successful application of hierarchical community models. First, classification schemes should be determined based on study objectives. For example, if the objective is to evaluate how habitat changes may disparately affect ground versus canopy nesting bird guilds, then use of a nest classification scheme would arguably be most appropriate. When interest is focused on estimating richness and understanding the community as a whole, our results suggest that use of an all-species grouping will be most prudent. Using an all-species grouping may be the best option when interest is focused on understanding species-level responses. Although this approach may lead to higher BCIs for parameters and thus greater uncertainty in the effects of covariates, it is less likely to lead to misleading results, which could be possible if species where misclassified relative to a specific covariate response. Second, some form of model selection to quantitatively compare different classification approaches should be used when study objectives call for use of multiple classification schemes. Model selection among hierarchical models is an area of active research. We presented results using a recently developed AUC approach that both accounts for imperfect detection of species and explicitly quantifies uncertainty (by calculating a BCI for AUC values). However, many other formal approaches to model selection exist (e.g., information-theoretic approaches, reversible jump MCMC). One new approach is to allow group membership to be estimated while including prior information about possible groupings, such that the model searches over all possible combinations and gives posterior weight to the best group for species placement relative to the set of covariates. This is an interesting and active area of research (Madon et al. 2013) but could present challenges to implementation for complex communities. Finally, it is a good idea to assess the sensitivity of results to different classification approaches when possible. If a sensitive or highly specialized species is placed in a group that also contains a ubiquitous species that is less sensitive to environmental change or management actions, the resulting inferences can be misleading. Species- and group-specific parameter estimates can be compared across multiple classification schemes when study objectives include the need for precise species-level estimates. Covariate relationships can be honed in on for individual species if parameter estimates are similar across multiple classifications. Conversely, if species-level estimates are quite different (and have nonoverlapping BCIs) with different classifications, then it is clear that such estimates are not meaningful (Picard et al. 2012). One indication of a poor match between a species and group is imprecise estimates even when the total number of detections for the group is high.

Classifying species into groups, coupled with multispecies hierarchical models, provides an opportunity to examine the influence of macroecological covariates and climate change on species assemblages at large spatial scales (e.g., Ruiz-Gutiérrez et al. 2010; Ruiz-Gutiérrez and Zipkin 2011). These approaches gain importance because emerging conservation paradigms and strategies favor assessments of multispecies responses across heterogeneous landscapes (Caro 2010). We stress the importance of clearly defined objectives and hypotheses before embarking in studies of this complexity. Our analysis should help researchers to understand the potential trade-offs with different classification groupings.

## Supporting Information

Figure S1 Total number of detections by species for the Puerto Rico and Hudson River Valley data sets.

Figure S2 Posterior mean estimates of species richness with 95% posterior credible intervals for the full community model and each of the three different classification approaches for the Puerto Rico (top) and Hudson River Valley (bottom) data sets at each site.

Figure S3 Individual species occurrence probabilities (posterior means with 95% credible intervals) in the four different habitat types (Agriculture, Forest, Urban, and Edge) of the study area in southwestern Puerto Rico for four important species (ANMA, Antillean mango, *Anthra- cothorax dominicus;* PRVI, Puerto Rican vireo, *Vireo latimeri;* PRSP, Puerto Rican spindalis, *Spindalis portoricensis;* and PUEB, Puerto Rican bullfinch, *Loxigilla portoricensis).*

Figure S4 Mean marginal occurrence probabilities for four priority conservation species (VESP, Vesper sparrow, *Pooecetes gramineus;* CERW, Cerulean warbler, *Setophaga cerulean*; BTBW, Black-throated blue warbler, *Setophaga caerulescens*; and SCTA, Scarlet tanager, *Piranga olivacea*) in relation to forest fragment area in the Hudson River Valley.

Figure S5 Posterior mean occurrence probabilities for four priority conservation species (Vesper sparrow, VESP: two detections at two sites; Cerulean warbler, CERW: seven detections at six sites; Black-throated blue warbler, BTBW: 20 detections at nine sites; and Scarlet tanager, SCTA: 141 detections at 56 sites) at individual sites in the Hudson River Valley study area.

Table S1 Individual observed species and their group classifications (PR: H – Habitat, M – Microhabitat, D – Diet; Hudson River Valley: Nest location – N, Habitat – H, D – Diet, see Table 1) for both the Puerto Rico and Hudson River Valley data sets.

**Table S2.** Model performance for each group classification approach (italics and bold; PR: H – Habitat, M – Microhabitat, D – Diet; Hudson River Valley: Nest location – N, Habitat – H, D – Diet, see Table 1) and subgroup within each group classification approach calculated by computing the area under the curve of the receiver operating characteristic (AUC) for both the Puerto Rico and Hudson River Valley data sets. **Data S1.** WinBUGS code and convergence assessment.
