# Supplementary figures and images for: Guidelines for a priori grouping of species in hierarchical community models
Source: Ecol Evol. 2014 Feb 22;4(7):877–88. doi: 10.1002/ece3.976 (PMC3997306; doi:10.1002/ece3.976)

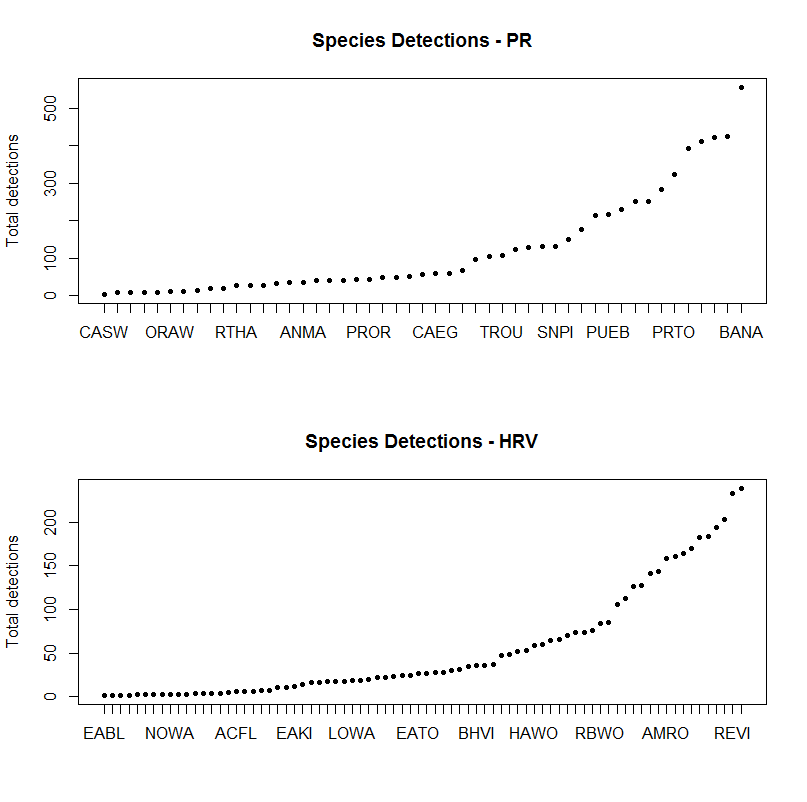

Supplement: Figure S1 — Total number of detections by species for the Puerto Rico and Hudson River Valley data sets. [file ece30004-0877-sd1.tiff]

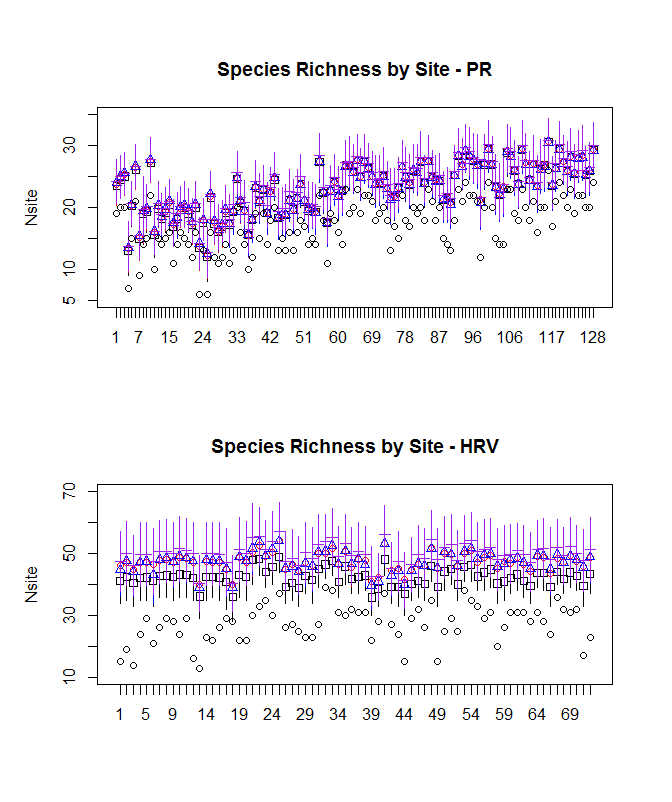

Supplement: Figure S2 — Posterior mean estimates of species richness with 95% posterior credible intervals for the full community model and each of the three different classification approaches for the Puerto Rico (top) and Hudson River Valley (bottom) data sets at each site. [file ece30004-0877-sd2.tiff]

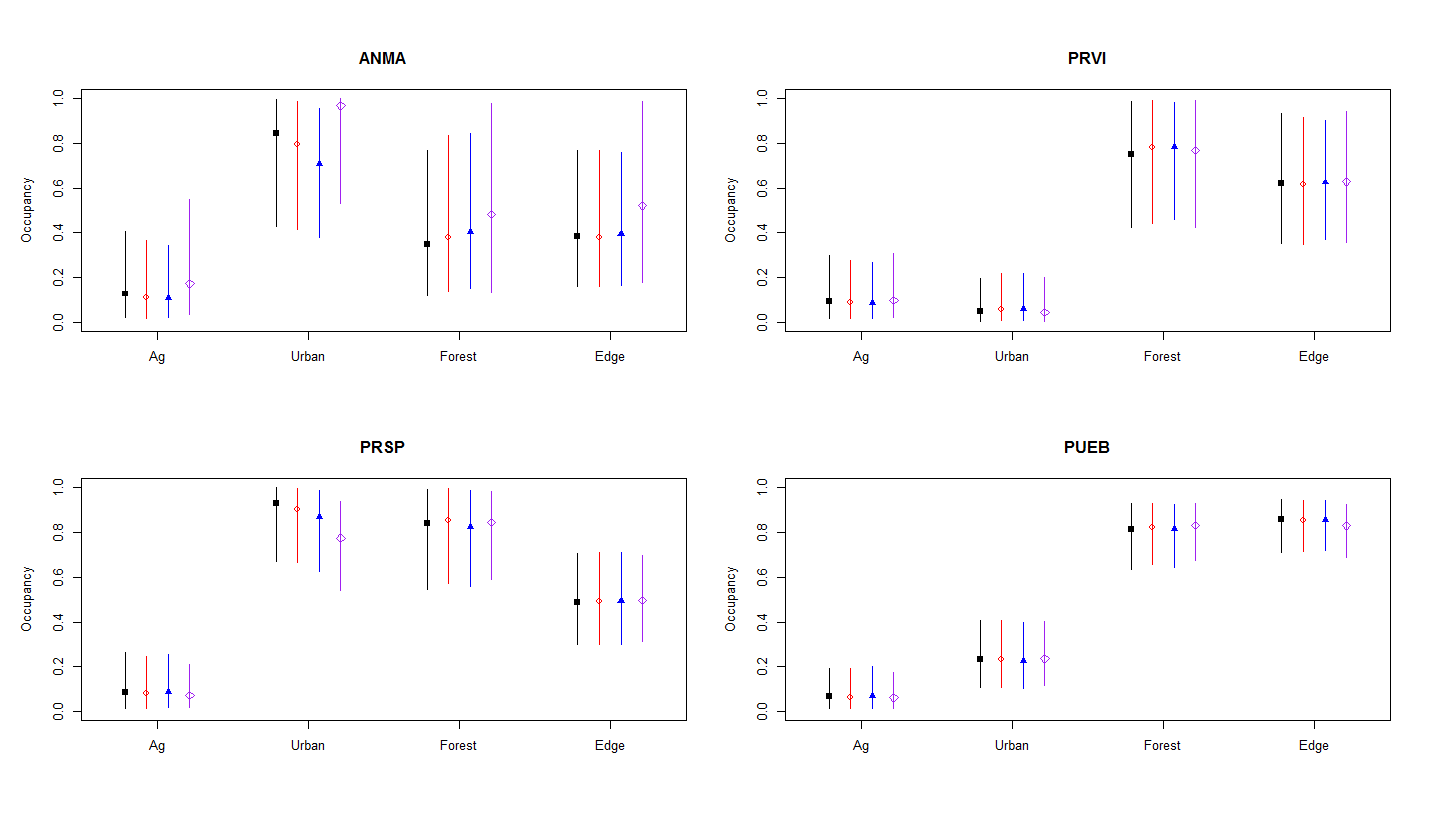

Supplement: Figure S3 — Individual species occurrence probabilities (posterior means with 95% credible intervals) in the four different habitat types (Agriculture, Forest, Urban, and Edge) of the study area in southwestern Puerto Rico for four important species (ANMA, Antillean mango, Anthra- cothorax dominicus; PRVI, Puerto Rican vireo, Vireo latimeri; PRSP, Puerto Rican spindalis, Spindalis portoricensis; and PUEB, Puerto Rican bullfinch, Loxigilla portoricensis). [file ece30004-0877-sd3.tiff]

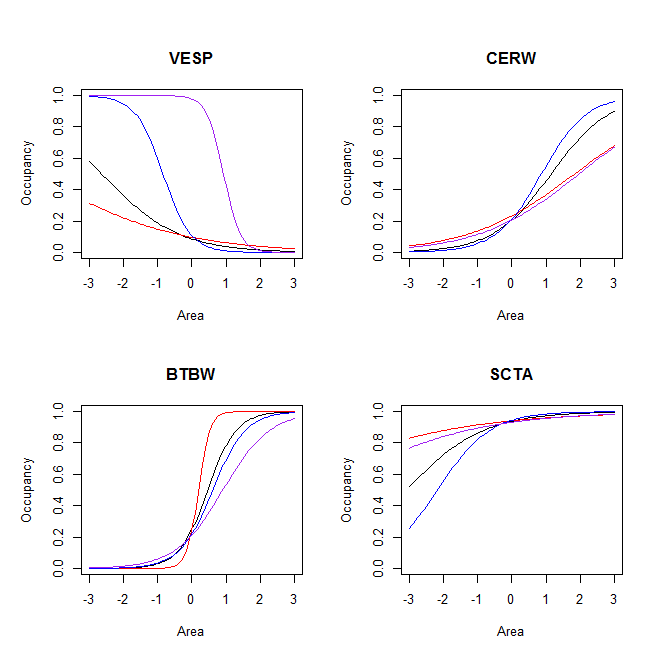

Supplement: Figure S4 — Mean marginal occurrence probabilities for four priority conservation species (VESP, Vesper sparrow, Pooecetes gramineus; CERW, Cerulean warbler, Setophaga cerulean; BTBW, Black-throated blue warbler, Setophaga caerulescens; and SCTA, Scarlet tanager, Piranga olivacea) in relation to forest fragment area in the Hudson River Valley. [file ece30004-0877-sd4.tiff]

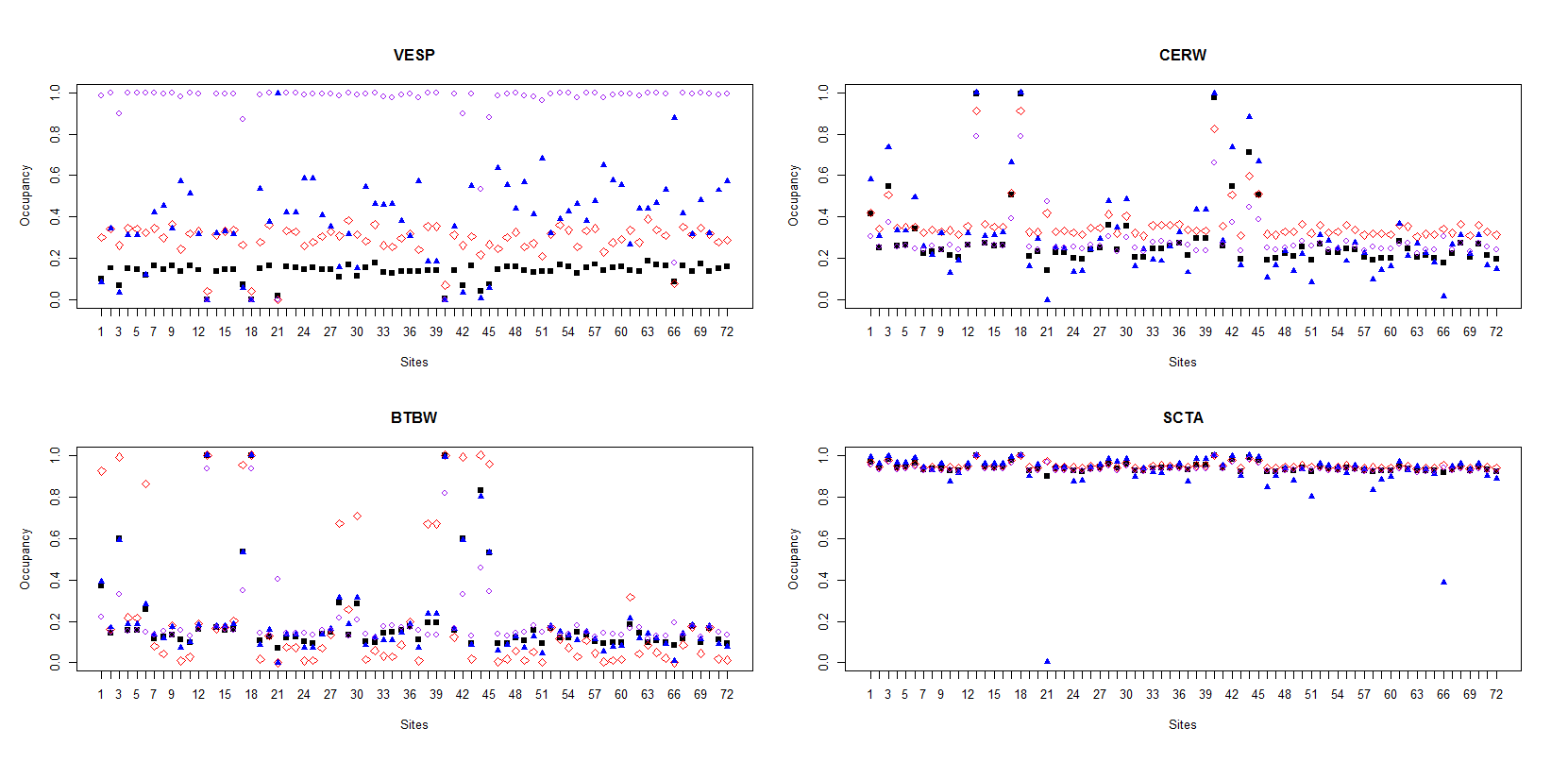

Supplement: Figure S5 — Posterior mean occurrence probabilities for four priority conservation species (Vesper sparrow, VESP: two detections at two sites; Cerulean warbler, CERW: seven detections at six sites; Black-throated blue warbler, BTBW: 20 detections at nine sites; and Scarlet tanager, SCTA: 141 detections at 56 sites) at individual sites in the Hudson River Valley study area. [file ece30004-0877-sd5.tiff]
