# Supplementary material for: Guidelines for a priori grouping of species in hierarchical community models
Source: Ecol Evol. 2014 Feb 22;4(7):877–88. doi: 10.1002/ece3.976 (PMC3997306; doi:10.1002/ece3.976)
Supplement: Table S1 — Individual observed species and their group classifications (PR: H – Habitat, M – Microhabitat, D – Diet; Hudson River Valley: Nest location – N, Habitat – H, D – Diet, see Table 1) for both the Puerto Rico and Hudson River Valley data sets. [file ece30004-0877-sd6.docx]

Supplement Table 1. Individual observed species and their group classifications (PR: H- Habitat, M – Microhabitat, D – Diet; HRV: Nest location – N, Habitat – H, D – Diet, see Table 1) for both the Puerto Rico and Hudson River Valley data sets.

| **Puerto Rico Data Set** | | | | | |
| --- | --- | --- | --- | --- | --- |
| **Species** | **Common Name** | **Scientific Name** | **Habitat** | **Microhabitat** | **Diet** |
| ADWA | Adelaide's Warbler | *Dendroica adelaidae* | Coastal Forest | Interspersed Forest | Insectivore |
| AFCD | African Collared Dove | *Streptopelia roseogrisea* | Coastal Forest | Open | Granivore |
| AMKE | American Kestrel | *Falco sparverius* | Coastal Forest | Open | Carnivore |
| ANEU | Antillean Euphonia | *Euphonia musica* | Wet, Upper Forests | Dense Forest | Frugivore |
| ANMA | Antillean Mango | *Anthracothorax dominicus* | Wet, Upper Forests | Dense Forest | Nectarivore |
| BANA | Bananaquit | *Coereba flaveola* | Wet, Upper Forests | Open | Nectarivore |
| BFGR | Black Faced Grassquit | *Tiaris bicolor* | Open/Anthropogenic | Grassland | Granivore |
| BWVI | Black-Whiskered Vireo | *Vireo altiloquus* | Wet, Upper Forests | Interspersed Forest | Insectivore |
| BRMA | Bronze Mannikin | *Lonchura cucullata* | Open/Anthropogenic | Grassland | Granivore |
| CAEL | Caribbean Elaenia | *Elaenia martinica* | Coastal Forest | Interspersed Forest | Insectivore |
| CAEG | Cattle Egret | *Bulbulcus ibis* | Open/Anthropogenic | Grassland | Insectivore |
| CASW | Cave Swallow | *Petrochelidon fulva* | Coastal Forest | Open | Insectivore |
| COGD | Common Ground Dove | *Columbina passerina* | Coastal Forest | Grassland | Granivore |
| GRSP | Grasshopper Sparrow | *Ammodramus savannarum* | Open/Anthropogenic | Grassland | Omnivore |
| GRAK | Gray Kingbird | *Tyrannus dominicensis* | Coastal Forest | Open | Insectivore |
| GREG | Great Egret | *Ardea alba* | Open/Anthropogenic | Grassland | Omnivore |
| GAGR | Greater Antillean Grackle | *Quiscalus niger* | Open/Anthropogenic | Open | Omnivore |
| HOSP | House Sparrow | *Passer domesticus* | Open/Anthropogenic | Open | Granivore |
| INSI | Indian Silverbill | *Euodice malabarica* | Open/Anthropogenic | Grassland | Granivore |
| KWQD | Key West Quail-Dove | *Geotrygon chrysia* | Coastal Forest | Dense Forest | Granivore |
| LAPE | Lesser Antillean Peewee | *Contopus latirostris* | Coastal Forest | Interspersed Forest | Insectivore |
| LOKI | Loggerhead Kingbird | *Tyrannus caudifasciatus* | Wet, Upper Forests | Dense Forest | Insectivore |
| MACU | Mangrove Cuckoo | *Coccyzus minor* | Coastal Forest | Interspersed Forest | Omnivore |
| MODO | Mourning Dove | *Zenaida macrocura* | Coastal Forest | Open | Insectivore |
| NOMO | Northern Mockingbird | *Mimus polyglottos* | Coastal Forest | Open | Omnivore |
| NUMA | Nutmeg Mannikin | *Lonchura punctulata* | Open/Anthropogenic | Grassland | Granivore |
| ORBI | Orange Bishop | *Euplectes franciscanus* | Open/Anthropogenic | Grassland | Granivore |
| ORAW | Orange Cheeked Waxbill | *Estrilda melpoda* | Open/Anthropogenic | Grassland | Granivore |
| PETH | Pearly-Eyed Thrasher | *Margarops fuscatus* | Coastal Forest | Open | Omnivore |
| PTWH | Pin-Tailed Whydah | *Vidua macrocura* | Open/Anthropogenic | Grassland | Granivore |
| PUEB | Puerto Rican Bullfinch | *Loxigilla portoricensis* | Wet, Upper Forests | Interspersed Forest | Frugivore |
| PREM | Puerto Rican Emerald | *Chlorostilbon maugaeus* | Wet, Upper Forests | Dense Forest | Nectarivore |
| PRFL | Puerto Rican Flycatcher | *Myiarchus antillarum* | Coastal Forest | Open | Insectivore |
| PRLC | Puerto Rican Lizard Cuckoo | *Coccyzus vieilloti* | Coastal Forest | Dense Forest | Carnivore |
| PROR | Puerto Rican Oriole | *Icterus dominicensis* | Wet, Upper Forests | Dense Forest | Insectivore |
| PRSP | Puerto Rican Spindalis | *Spindalis portoricensis* | Wet, Upper Forests | Interspersed Forest | Frugivore |
| PRTO | Puerto Rican Tody | *Todus mexicanus* | Coastal Forest | Dense Forest | Insectivore |
| PRVI | Puerto Rican Vireo | *Vireo latimeri* | Wet, Upper Forests | Dense Forest | Insectivore |
| PRWO | Puerto Rican Woodpecker | *Melanerpes portoricensis* | Wet, Upper Forests | Interspersed Forest | Insectivore |
| RLTH | Red-Legged Thrush | *Turdus plumbeus* | Wet, Upper Forests | Dense Forest | Frugivore |
| RTHA | Red-Tailed Hawk | *Buteo jamaicensis* | Wet, Upper Forests | Open | Carnivore |
| ROPI | Rock Pigeon | *Columba livia* | Wet, Upper Forests | Open | Omnivore |
| RUQD | Ruddy Quail-Dove | *Geotrygon montana* | Coastal Forest | Open | Granivore |
| SNPI | Scaly-Naped Pigeon | *Patagioenas squamosa* | Wet, Upper Forests | Dense Forest | Frugivore |
| SHCO | Shiny Cowbird | *Molothrus bonariensis* | Open/Anthropogenic | Open | Omnivore |
| SBAN | Smooth-Billed Ani | *Crotophaga ani* | Coastal Forest | Open | Omnivore |
| TROU | Troupial | *Icterus icterus* | Coastal Forest | Open | Omnivore |
| WWDO | White-Winged Dove | *Zenaida asiatica* | Coastal Forest | Open | Granivore |
| YFGR | Yellow-Faced Grassquit | *Tiaris olivaceus* | Open/Anthropogenic | Grassland | Granivore |
| ZEND | Zenaida Dove | *Zenaida aurita* | Coastal Forest | Open | Granivore |

| **Hudson River Valley Data Set** | | | | | |
| --- | --- | --- | --- | --- | --- |
| **Species** | **Common Name** | **Scientific Name** | **Nest** | **Habitat** | **Diet** |
| ACFL | Acadian Flycatcher | *Empidonax virescens* | Tree | Arboreal | Insectivore |
| AMCR | American Crow | *Corvus brachyrhynchos* | Tree | Terrestrial | Omnivore |
| AMGO | American Goldfinch | *Carduelis tristis* | Tree | Bush | Granivore |
| AMRE | American Redstart | *Setophaga ruticilla* | Tree | Arboreal | Insectivore |
| AMRO | American Robin | *Turdus migratorius* | Bush | Terrestrial | Frugivore |
| BAOR | Baltimore Oriole | *Icterus galbula* | Tree | Tall vegetation | Frugivore |
| BASW | Barn Swallow | *Hirundo rustica* | Ledge | Open Air | Insectivore |
| BBWA | Bay-breasted Warbler | *Dendroica castanea* | Tree | Arboreal | Insectivore |
| BAWW | Black-and-White Warbler | *Mniotilta varia* | Ground | Tree trunk | Insectivore |
| BBCU | Black-billed Cuckoo | *Coccyzus erythropthalmus* | Bush | Arboreal | Insectivore |
| BCCH | Black-capped Chickadee | *Poecile atricapillus* | Cavity | Arboreal | Insectivore |
| BPWA | Blackpoll Warbler | *Setophaga striata* | Tree | Arboreal | Insectivore |
| BTBW | Black-throated Blue Warbler | *Dendroica caerulescens* | Bush | Arboreal | Insectivore |
| BTNW | Black-throated Green Warbler | *Setophaga virens* | Tree | Arboreal | Insectivore |
| BLJA | Blue Jay | *Cyanocitta cristata* | Tree | Arboreal | Omnivore |
| BGGN | Blue-gray Gnatcatcher | *Polioptila caerulea* | Tree | Arboreal | Insectivore |
| BHVI | Blue-headed Vireo | *Vireo solitarius* | Bush | Arboreal | Insectivore |
| BWWA | Blue-winged Warbler | *Vermivora pinus* | Ground | Bush | Insectivore |
| BRCR | Brown Creeper | *Certhia americana* | Cavity | Tree trunk | Insectivore |
| BRTH | Brown Thrasher | *Toxostoma rufum* | Bush | Ground/shrub | Omnivore |
| BHCO | Brown-headed Cowbird | *Molothrus ater* | Bush | Terrestrial | Omnivore |
| CAWA | Canada Warbler | *Wilsonia canadensis* | Ground | Bush | Insectivore |
| CAWR | Carolina Wren | *Thryothorus ludovicianus* | Cavity | Ground/shrub | Insectivore |
| CEDW | Cedar Waxwing | *Bombycilla cedrorum* | Tree | Tall vegetation | Frugivore |
| CERW | Cerulean Warbler | *Dendroica cerulea* | Tree | Arboreal | Insectivore |
| CSWA | Chestnut-sided Warbler | *Dendroica pensylvanica* | Bush | Bush | Insectivore |
| CHIP | Chipping Sparrow | *Spizella passerina* | Bush | Terrestrial | Granivore |
| COGR | Common Grackle | *Quiscalus quiscula* | Bush | Terrestrial | Granivore |
| CORA | Common Raven | *Corvus corax* | Ledge | Terrestrial | Omnivore |
| COYE | Common Yellowthroat | *Geothlypis trichas* | Ground | Bush | Insectivore |
| DOWO | Downy Woodpecker | *Picoides pubescens* | Cavity | Tree trunk | Insectivore |
| EABL | Eastern Bluebird | *Sialia sialis* | Cavity | Terrestrial | Insectivore |
| EAKI | Eastern Kingbird | *Tyrannus tyrannus* | Tree | Open Air | Insectivore |
| EAPH | Eastern Phoebe | *Sayornis phoebe* | Ledge | Open Air | Insectivore |
| EATO | Eastern Towhee | *Pipilo erythrophthalmus* | Bush | Terrestrial | Omnivore |
| EWPE | Eastern Wood-pewee | *Contopus virens* | Tree | Arboreal | Insectivore |
| EUST | European Starling | *Sturnus vulgaris* | Cavity | Terrestrial | Omnivore |
| FICR | Fish Crow | *Corvus ossifragus* | Tree | Terrestrial | Omnivore |
| GRCA | Gray Catbird | *Dumetella carolinensis* | Bush | Bush | Insectivore |
| GCFL | Great Crested Flycatcher | *Myiarchus crinitus* | Cavity | Arboreal | Insectivore |
| HAWO | Hairy Woodpecker | *Picoides villosus* | Cavity | Tree trunk | Insectivore |
| HETH | Hermit Thrush | *Catharus guttatus* | Ground | Terrestrial | Insectivore |
| HOWA | Hooded Warbler | *Wilsonia citrina* | Bush | Tall vegetation | Insectivore |
| HOWR | House Wren | *Troglodytes aedon* | Cavity | Bush | Insectivore |
| LEFL | Least Flycatcher | *Empidonax minimus* | Tree | Arboreal | Insectivore |
| LOWA | Louisiana Waterthrush | *Seiurus motacilla* | Ground | Terrestrial | Insectivore |
| MAWA | Magnolia Warbler | *Dendroica magnolia* | Bush | Tall vegetation | Insectivore |
| MODO | Mourning Dove | *Zenaida macroura* | Bush | Terrestrial | Granivore |
| NAWA | Nashville Warbler | *Vermivora ruficapilla* | Ground | Bush | Insectivore |
| NOCA | Northern Cardinal | *Cardinalis cardinalis* | Bush | Ground/shrub | Omnivore |
| NOFL | Northern Flicker | *Colaptes auratus* | Cavity | Tree trunk | Insectivore |
| NOMO | Northern Mockingbird | *Mimus polyglottos* | Bush | Ground/shrub | Omnivore |
| NOPA | Northern Parula | *Parula americana* | Tree | Arboreal | Insectivore |
| NOWA | Northern Waterthrush | *Seiurus noveboracensis* | Ground | Terrestrial | Insectivore |
| OVEN | Ovenbird | *Seiurus aurocapilla* | Ground | Terrestrial | Insectivore |
| PIWO | Pileated Woodpecker | *Dryocopus pileatus* | Cavity | Tree trunk | Insectivore |
| PISI | Pine Siskin | *Carduelis pinus* | Tree | Arboreal | Granivore |
| PIWA | Pine Warbler | *Dendroica pinus* | Tree | Arboreal | Insectivore |
| RBWO | Red-bellied Woodpecker | *Melanerpes carolinus* | Cavity | Tree trunk | Insectivore |
| RBNU | Red-breasted Nuthatch | *Sitta canadensis* | Cavity | Tree trunk | Insectivore |
| REVI | Red-eyed Vireo | *Vireo olivaceus* | Tree | Arboreal | Insectivore |
| RWBL | Red-winged Blackbird | *Agelaius phoeniceus* | Bush | Bush | Omnivore |
| RBGR | Rose-breasted Grosbeak | *Pheucticus ludovicianus* | Bush | Arboreal | Omnivore |
| SCTA | Scarlet Tanager | *Piranga olivacea* | Tree | Arboreal | Insectivore |
| SOSP | Song Sparrow | *Melospiza melodia* | Ground | Terrestrial | Granivore |
| TRSW | Tree Swallow | *Tachycineta bicolor* | Cavity | Open Air | Insectivore |
| TUTI | Tufted Titmouse | *Baeolophus bicolor* | Cavity | Arboreal | Omnivore |
| VEER | Veery | *Catharus fuscescens* | Ground | Terrestrial | Insectivore |
| VESP | Vesper Sparrow | *Pooecetes gramineus* | Ground | Terrestrial | Granivore |
| WBNU | White-breasted Nuthatch | *Sitta carolinensis* | Cavity | Tree trunk | Insectivore |
| WIWR | Winter Wren | *Troglodytes troglodytes* | Ground | Terrestrial | Insectivore |
| WOTH | Wood Thrush | *Hylocichla mustelina* | Bush | Terrestrial | Insectivore |
| WEWA | Worm-eating Warbler | *Helmitheros vermivorum* | Ground | Ground/shrub | Insectivore |
| YEWA | Yellow Warbler | *Setophaga petechia* | Bush | Bush | Insectivore |
| YBSA | Yellow-bellied Sapsucker | *Sphyrapicus varius* | Cavity | Tree trunk | Omnivore |
| YBCU | Yellow-billed Cuckoo | *Coccyzus americanus* | Tree | Arboreal | Insectivore |
| YRWA | Yellow-rumped Warbler | *Dendroica coronata* | Tree | Tall vegetation | Insectivore |
| YTVI | Yellow-throated Vireo | *Vireo flavifrons* | Tree | Arboreal | Insectivore |

Supplement Table 2. Model performance for each group classification approach (italics and bold; PR: H- Habitat, M – Microhabitat, D – Diet; HRV: Nest location – N, Habitat – H, D – Diet, see Table 1) and subgroup within each group classification approach calculated by computing the area under the curve of the receiver operating characteristic (AUC) for both the Puerto Rico and Hudson River Valley data sets.

| **HRV** | **Mean** | **2.50%** | **97.50%** |
| --- | --- | --- | --- |
| ***All species*** | ***0.84*** | ***0.77*** | ***0.90*** |
| ***Nest Location*** | ***0.75*** | ***0.52*** | ***0.91*** |
| Bush-nest | 0.83 | 0.71 | 0.94 |
| Cavity | 0.68 | 0.50 | 0.84 |
| Ground-nest | 0.77 | 0.64 | 0.88 |
| Ledge | 0.67 | 0.39 | 0.89 |
| Tree-nest | 0.77 | 0.65 | 0.88 |
| ***Habitat*** | ***0.77*** | ***0.54*** | ***0.96*** |
| Arboreal | 0.78 | 0.65 | 0.90 |
| Bush | 0.73 | 0.57 | 0.87 |
| Ground/shrub | 0.76 | 0.53 | 0.94 |
| Open Air | 0.77 | 0.51 | 0.98 |
| Terrestrial | 0.84 | 0.71 | 0.95 |
| Tree trunk | 0.68 | 0.48 | 0.91 |
| Vegetation | 0.82 | 0.59 | 0.98 |
| ***Diet*** | ***0.79*** | ***0.57*** | ***0.93*** |
| Frugivore | 0.74 | 0.47 | 0.93 |
| Granivore | 0.77 | 0.55 | 0.97 |
| Insectivore | 0.85 | 0.77 | 0.91 |
| Omnivore | 0.77 | 0.61 | 0.91 |
| **PR** | **Mean** | **2.50%** | **97.50%** |
| ***All species*** | ***0.80*** | ***0.76*** | ***0.85*** |
| ***Habitat*** | ***0.75*** | ***0.68*** | ***0.82*** |
| Wet, Upper Forests | 0.75 | 0.68 | 0.81 |
| Open/Anthropogenic | 0.76 | 0.68 | 0.84 |
| Coastal Forest | 0.75 | 0.68 | 0.81 |
| ***Microhabitat*** | ***0.74*** | ***0.65*** | ***0.82*** |
| Dense Forest | 0.73 | 0.66 | 0.80 |
| Grassland | 0.74 | 0.65 | 0.83 |
| Interspersed Forest | 0.73 | 0.63 | 0.81 |
| Open | 0.76 | 0.70 | 0.83 |
| ***Diet*** | ***0.71*** | ***0.54*** | ***0.82*** |
| Carnivores | 0.63 | 0.47 | 0.78 |
| Frugivores | 0.72 | 0.64 | 0.80 |
| Granivores | 0.76 | 0.67 | 0.83 |
| Insectivores | 0.75 | 0.67 | 0.82 |
| Nectarivores | 0.67 | 0.53 | 0.79 |
| Omnivores | 0.74 | 0.64 | 0.83 |


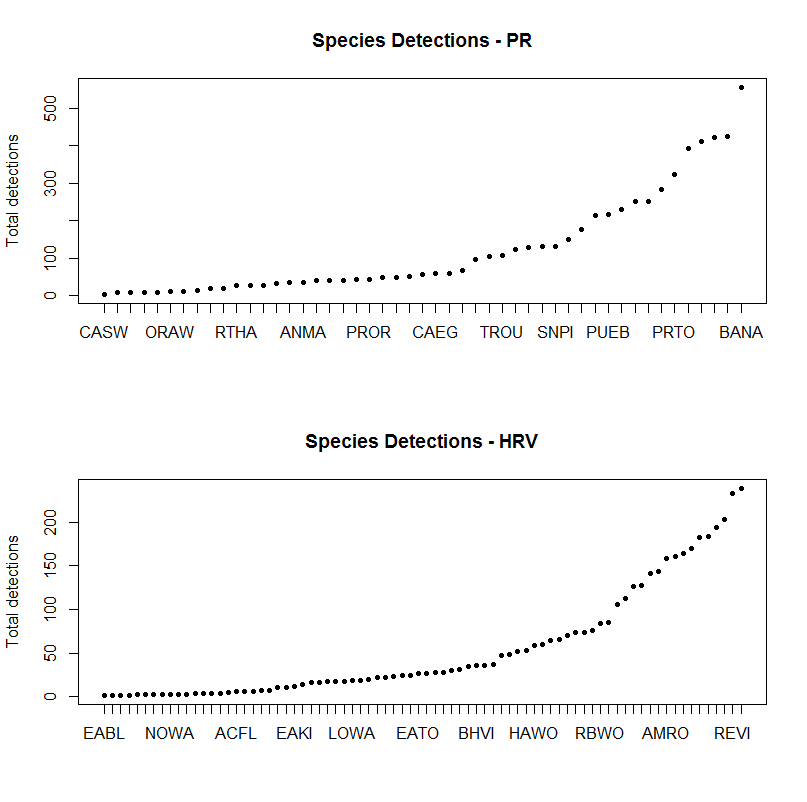


Supplement Figure 1. Total number of detections by species for the Puerto Rico and Hudson River Valley data sets.


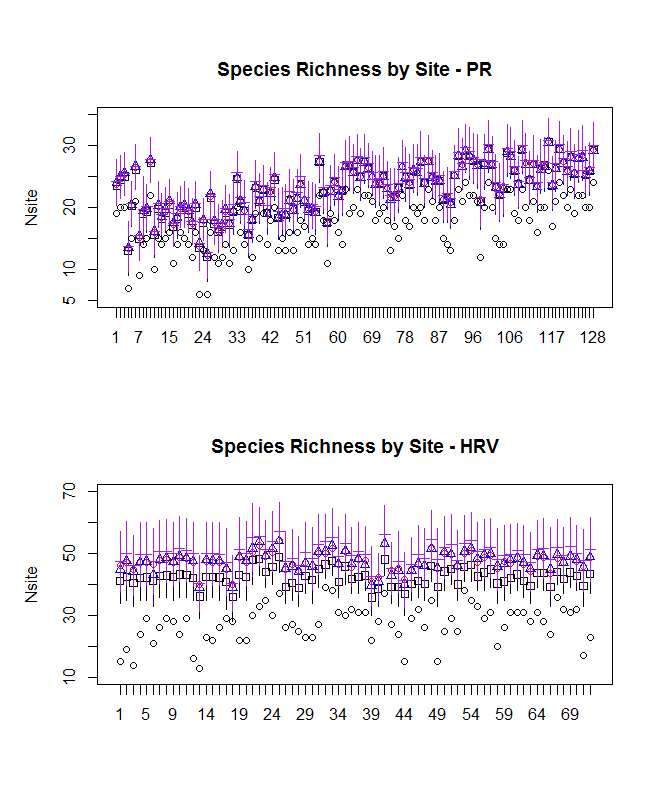


Supplement figure 2. Posterior mean estimates of species richness with 95% posterior credible intervals for the full community model and each of the three different classification approaches for the Puerto Rico (top) and Hudson River Valley (bottom) data sets at each site. Black circles represent observed number of species at each site and black squares represent using all of the species together in the full model. Red circles represent Habitat (Puerto Rico data set) and Nest location (Hudson River Valley data set), respectively; blue triangles represent Microhabitat (Puerto Rico data set) and Habitat (Hudson River Valley data set), and purple lines represent Diet for both data sets.

**
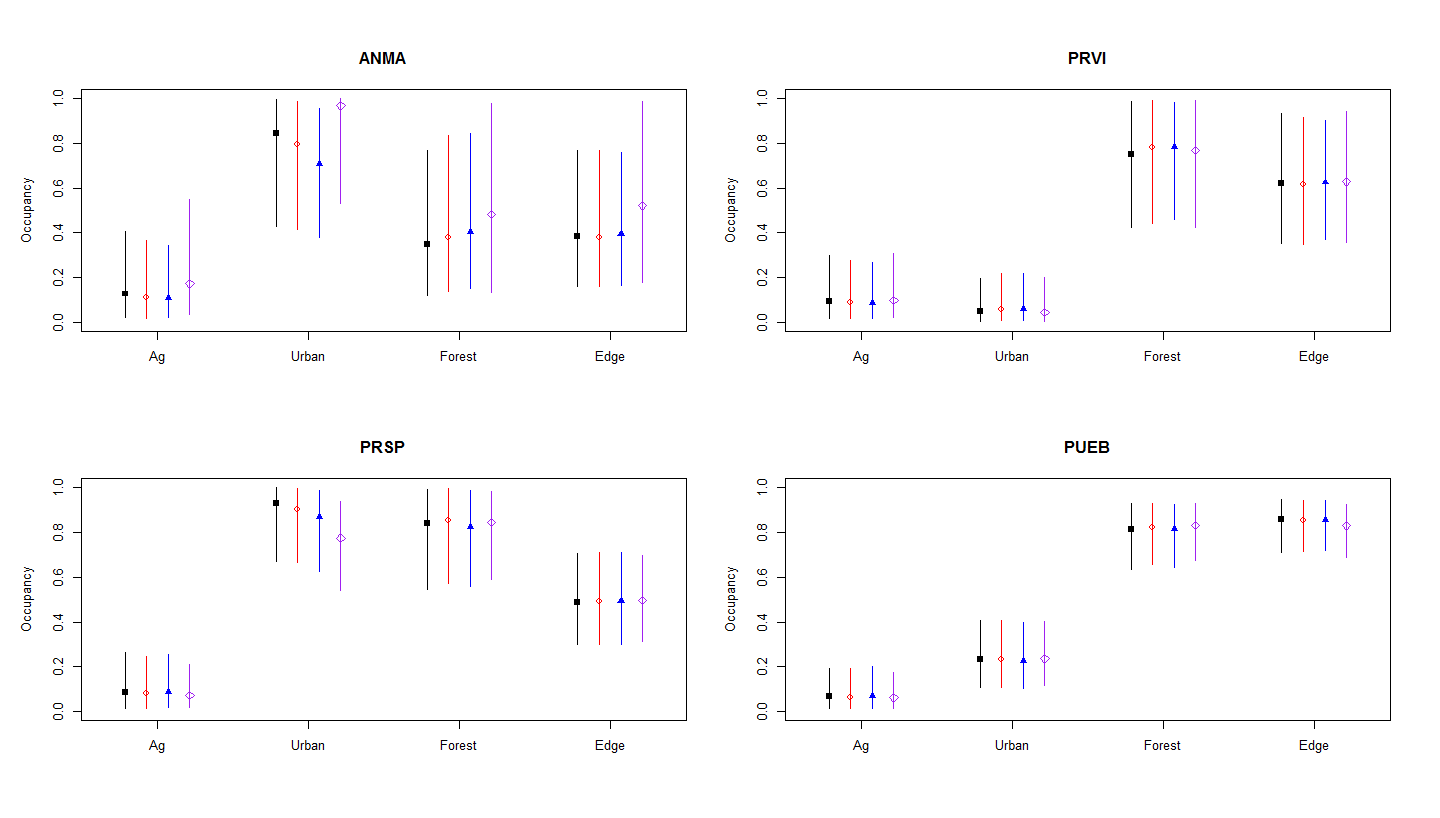
**

Supplement figure 3. Individual species occurrence probabilities (posterior means with 95% credible intervals) in the four different habitat types (Agriculture, Forest, Urban, and Edge) of the study area in southwestern Puerto Rico for four important species (ANMA: Antillean mango, *Anthracothorax dominicus*; PRVI: Puerto Rican vireo, *Vireo latimeri*; PRSP: Puerto Rican spindalis, *Spindalis portoricensis*; and PUEB: Puerto Rican bullfinch, *Loxigilla portoricensis*). We used three different classification schemes to group species: Habitat – red, Microhabitat – blue, and Diet – purple, along with using all of the species – black.


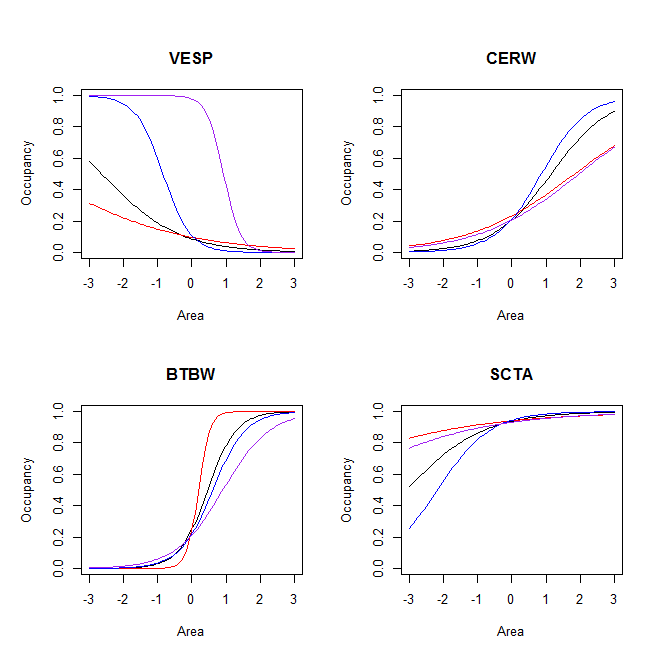


Supplement figure 4. Mean marginal occurrence probabilities for four priority conservation species (VESP: Vesper sparrow, *Pooecetes gramineus*; CERW: Cerulean warbler, *Setophaga cerulean*; BTBW: Black-throated blue warbler, *Setophaga caerulescens*; and SCTA: Scarlet tanager, *Piranga olivacea*) in relation to forest fragment area in the Hudson River Valley. We explored three different classification schemes for grouping species: Nest location – red lines, Habitat – blue lines, and Diet – purple lines; black lines represent using all of the species. Covariates have been standardized to have a mean of zero and variance of one. Although uncertainty was high for all models, using all species had the lowest uncertainty (narrowest credible intervals) followed by nest location, habitat, and diet models.


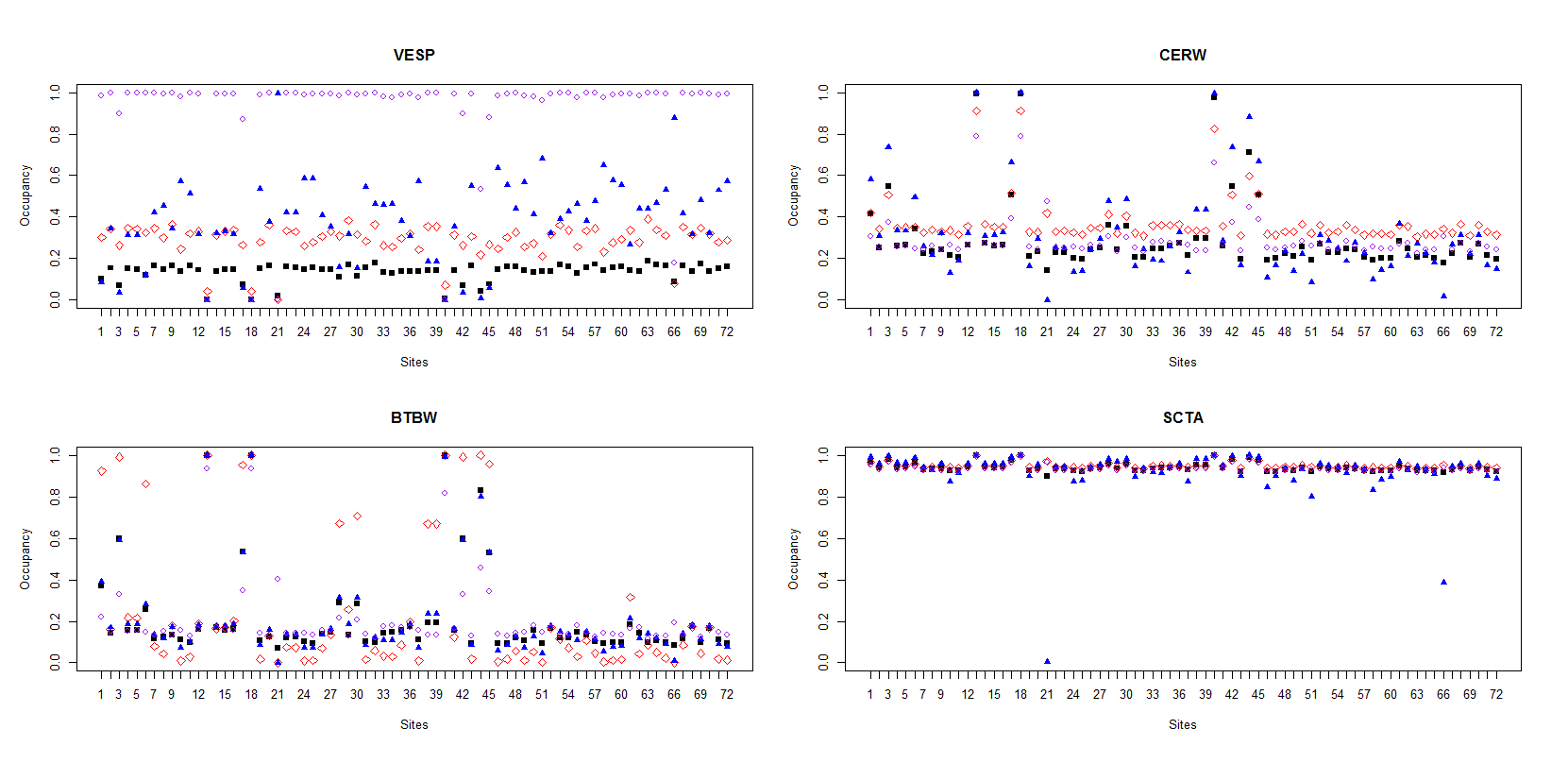


Supplement figure 5. Posterior mean occurrence probabilities for four priority conservation species (Vesper sparrow, VESP: 2 detections at 2 sites; Cerulean warbler, CERW: 7 detections at 6 sites; Black-throated blue warbler, BTBW: 20 detections at 9 sites; and Scarlet tanager, SCTA: 141 detections at 56 sites) at individual sites in the Hudson River Valley study area. We used three different classification schemes for grouping species: Nest location – red large diamonds, Habitat – blue triangles, and Diet – purple small diamonds; black squares represent using all of the species.
