# Supplementary material for: Guidelines for a priori grouping of species in hierarchical community models
Source: Ecol Evol. 2014 Feb 22;4(7):877–88. doi: 10.1002/ece3.976 (PMC3997306; doi:10.1002/ece3.976)
Supplement: Supplementary file 7 — Table S2. Model performance for each group classification approach (italics and bold; PR: H – Habitat, M – Microhabitat, D – Diet; Hudson River Valley: Nest location – N, Habitat – H, D – Diet, see Table 1) and subgroup within each group classification approach calculated by computing the area under the curve of the receiver operating characteristic (AUC) for both the Puerto Rico and Hudson River Valley data sets. Data S1. WinBUGS code and convergence assessment. [file ece30004-0877-sd7.docx]

**Supplement 2. WinBUGS code and convergence assessment.**

We fit each of the models with the different classification schemes separately using a Bayesian approach in WinBUGS (Spiegelhalter et al. 2003) through R (R2WinBUGS; Sturtz et al. 2005) by running three parallel chains each of length > 77,000 with burn-in of at least 25,000 and thinning by 25. The model code, including the prior distributions, is presented below.

**Puerto Rico**

model {

omega ~ dunif(0,1)

for (xx in 1:ntype){

psi.mean[xx] ~ dunif(0,1)

alpha[xx] <- log(psi.mean[xx]) - log(1-psi.mean[xx])

}

theta.mean ~ dunif(0,1)

beta <- log(theta.mean) - log(1-theta.mean)

b5 ~ dnorm(0, 0.001)

tau.u ~ dgamma(0.1,0.1)

tau.v ~ dgamma(0.1,0.1)

sigma.u <- 1/sqrt(tau.u)

sigma.v <- 1/sqrt(tau.v)

for (i in 1:(n+nzeroes)) {

w[i] ~ dbin(omega, 1)

for (xx in 1:ntype){

phi[xx,i] ~ dnorm(alpha[xx],tau.u)I(-12,12)

}

eta[i] ~ dnorm(beta, tau.v)

for (j in 1:J) {

logit(psi[j,i]) <- phi[type[j],i]

mu.psi[j,i] <- psi[j,i]*w[i]

Z[j,i] ~ dbin(mu.psi[j,i], 1)

for (k in 1:K[j]) {

logit(theta[j,k,i]) <- eta[i] + b5*year[j,k]

mu.theta[j,k,i] <- theta[j,k,i]*Z[j,i]

X[j,k,i] ~ dbin(mu.theta[j,k,i], 1)

}

}

}

for(j in 1:J){

Nsite[j]<- inprod(Z[j,1:(n+nzeroes)],w[1:(n+nzeroes)])

}

n0 <- sum(w[(n+1):(n+nzeroes)])

N <- n + n0

}

**Hudson River Valley**

model {

omega ~ dunif(0,1)

psi.mean ~ dunif(0,1)

alpha <- log(psi.mean) - log(1-psi.mean)

theta.mean ~ dunif(0,1)

beta <- log(theta.mean) - log(1-theta.mean)

mub1 ~ dnorm(0, 0.001)

mub2 ~ dnorm(0, 0.001)

mub3 ~ dnorm(0, 0.001)

mub4 ~ dnorm(0, 0.001)

mub44 ~ dnorm(0, 0.001)

b5 ~ dnorm(0, 0.001)

tau.u ~ dgamma(0.1,0.1)

tau.v ~ dgamma(0.1,0.1)

tau.b1 ~ dgamma(0.1,0.1)

tau.b4 ~ dgamma(0.1,0.1)

tau.b2 ~ dgamma(0.1,0.1)

tau.b3 ~ dgamma(0.1,0.1)

tau.b44 ~ dgamma(0.1,0.1)

sigma.u <- 1/sqrt(tau.u)

sigma.v <- 1/sqrt(tau.v)

for (i in 1:(n+nzeroes)) {

w[i] ~ dbin(omega, 1)

phi[i] ~ dnorm(alpha, tau.u)

eta[i] ~ dnorm(beta, tau.v)

b1[i] ~ dnorm(mub1, tau.b1)

b4[i] ~ dnorm(mub4, tau.b4)

b2[i] ~ dnorm(mub2, tau.b2)

b3[i] ~ dnorm(mub3, tau.b3)

b44[i] ~ dnorm(mub44, tau.b44)

for (j in 1:J) {

logit(psi[j,i]) <- phi[i] + b1[i]*pa[j] + b2[i]*area1[j] + b3[i]*perm[j]

mu.psi[j,i] <- psi[j,i]*w[i]

Z[j,i] ~ dbin(mu.psi[j,i], 1)

for (k in 1:K[j]) {

logit(theta[j,k,i]) <- eta[i] + b5*year[j,k] + b4[i]*date1[j,k] + b44[i]*date2[j,k]

mu.theta[j,k,i] <- theta[j,k,i]*Z[j,i]

X[j,k,i] ~ dbin(mu.theta[j,k,i], 1)

}

}

}

for(j in 1:J){

Nsite[j]<- inprod(Z[j,1:(n+nzeroes)],w[1:(n+nzeroes)])

}

n0 <- sum(w[(n+1):(n+nzeroes)])

N <- n + n0

}

**Modeling correlation between occurrence and detection**

We explored modeling a correlation ($\rho)$ between occurrence and detection in the model by allowing $\mu_{i}$ and $\upsilon_{i}$ to be jointly distributed such that $\left[ u_{i},v_{i} | \Sigma\right]\sim N(0,\Sigma)$ where $(\sigma_{\mu}^{2}, \sigma_{\nu}^{2})$ are the variance components among species for occurrence and detection, respectively, and $\sigma_{\mu\nu}$ is the covariance of the 2 x 2 matrix $\Sigma$. We tried this for both datasets and for all of the classification schemes. For the majority of the models (including all of the models run with the PR dataset) there was either no support for the correlation (posterior means = 0 and credible intervals largely overlapping 0) or the models did not converge (3 chains, >200,000 iterations) determined by examining trace plots, correlations, and looking at the estimated Rhat which was always above 1.1. For the HRV dataset there were several models that did have an estimable correlation between occurrence and detection and in these cases those results were used although the effect on the parameter estimates or inferences compared to the models without correlation were minimal. The groups for the HRV dataset where correlation was included were: all of the species grouped together, Diet: omnivores, insectivores, Nest: tree, ground, bush, and Habitat: terrestrial, bush, arboreal. The model is included below.

model {

omega ~ dunif(0,1)

psi.mean ~ dunif(0,1)

alpha <- log(psi.mean) - log(1-psi.mean)

theta.mean ~ dunif(0,1)

beta <- log(theta.mean) - log(1-theta.mean)

mub1 ~ dnorm(0, 0.001)

mub2 ~ dnorm(0, 0.001)

mub3 ~ dnorm(0, 0.001)

mub4 ~ dnorm(0, 0.001)

mub44 ~ dnorm(0, 0.001)

b5 ~ dnorm(0, 0.001)

tau.u ~ dgamma(0.1,0.1)

tau.v ~ dgamma(0.1,0.1)

tau.b1 ~ dgamma(0.1,0.1)

tau.b4 ~ dgamma(0.1,0.1)

tau.b2 ~ dgamma(0.1,0.1)

tau.b3 ~ dgamma(0.1,0.1)

tau.b44 ~ dgamma(0.1,0.1)

rho ~ dunif(-1,1)

var.v <- tau.v /(1.-pow(rho,2))

sigma.u <- 1/sqrt(tau.u)

sigma.v <- 1/sqrt(tau.v)

for (i in 1:(n+nzeroes)) {

w[i] ~ dbin(omega, 1)

phi[i] ~ dnorm(alpha, tau.u)

mu.v[i] <- beta + (rho*sigma.v/sigma.u)*(phi[i] - alpha)

eta[i] ~ dnorm(mu.v[i], var.v)

b1[i] ~ dnorm(mub1, tau.b1)

b4[i] ~ dnorm(mub4, tau.b4)

b2[i] ~ dnorm(mub2, tau.b2)

b3[i] ~ dnorm(mub3, tau.b3)

b44[i] ~ dnorm(mub44, tau.b44)

for (j in 1:J) {

logit(psi[j,i]) <- phi[i] + b1[i]*pa[j] + b2[i]*area1[j] + b3[i]*perm[j]

mu.psi[j,i] <- psi[j,i]*w[i]

Z[j,i] ~ dbin(mu.psi[j,i], 1)

for (k in 1:K[j]) {

logit(theta[j,k,i]) <- eta[i] + b5*year[j,k] + b4[i]*date1[j,k] + b44[i]*date2[j,k]

mu.theta[j,k,i] <- theta[j,k,i]*Z[j,i]

X[j,k,i] ~ dbin(mu.theta[j,k,i], 1)

}

}

}

for(j in 1:J){

Nsite[j]<- inprod(Z[j,1:(n+nzeroes)],w[1:(n+nzeroes)])

}

n0 <- sum(w[(n+1):(n+nzeroes)])

N <- n + n0

}

**Convergence**

Convergence was assessed by using three different metrics. First, we used 3 chains running simultanesouly with starting points dispersed throughout the parameter space. Second, we explored variation between and within simulated sequences until these are roughly equal. Third, for MCMC chains that are inefficient (require a very large amount of computing time) we explored changing/altering priors and using different parameterizations of the same model. Additionally, we had a sufficiently large burn-in (at least 25,000 iterations) and thinned by 25 for each chain to potentially eliminate additional correlation. We monitored each parameter of interest and assessed the effective number of independent draws along with an estimated Rhat (Gelman and Rubin 1992) and discarded any runs where Rhat >1.09 and the effective number of independent draws < 500. We also looked at trace plots, correlation and the estimated variances of each parameter to get a better feel for how well our models were running.

Gelman, A. & Rubin, D.B. (1992) Inference from iterative simulation using multiple sequences.

*Statistical Science* 7, 457-511.
